# Micro- to Nanoscale Bio-Hybrid Hydrogels Engineered by Ionizing Radiation

**DOI:** 10.3390/biom11010047

**Published:** 2020-12-31

**Authors:** Clelia Dispenza, Daniela Giacomazza, Mats Jonsson

**Affiliations:** 1 Dipartimento di Ingegneria, Università degli Studi di Palermo, Viale delle Scienze 6, 90128 Palermo, Italy; 2 Istituto di BioFisica, Consiglio Nazionale delle Ricerche, Via U. La Malfa 153, 90146 Palermo, Italy; daniela.giacomazza@cnr.it; 3 Department of Chemistry, School of Engineering Sciences in Chemistry, Biotechnology and Health, KTH Royal Institute of Technology, SE-100 44 Stockholm, Sweden; matsj@kth.se

**Keywords:** radiation chemistry, micro-/nano-gel patterns, nanogels, bio-hybrid hydrogels, drug delivery, tissue engineering

## Abstract

Bio-hybrid hydrogels consist of a water-swollen hydrophilic polymer network encapsulating or conjugating single biomolecules, or larger and more complex biological constructs like whole cells. By modulating at least one dimension of the hydrogel system at the micro- or nanoscale, the activity of the biological component can be extremely upgraded with clear advantages for the development of therapeutic or diagnostic micro- and nano-devices. Gamma or e-beam irradiation of polymers allow a good control of the chemistry at the micro-/nanoscale with minimal recourse to toxic reactants and solvents. Another potential advantage is to obtain simultaneous sterilization when the absorbed doses are within the sterilization dose range. This short review will highlight opportunities and challenges of the radiation technologies to produce bio-hybrid nanogels as delivery devices of therapeutic biomolecules to the target cells, tissues, and organs, and to create hydrogel patterns at the nano-length and micro-length scales on surfaces.

## 1. Introduction

Hydrogels have emerged as an important class of functional materials due to their unique structure, tailorable functionalities, and properties, such as high water content, interconnected porosity, softness, and flexibility, that make them resemble biological materials like mucus or the extracellular matrix that surrounds cells, tissues, organs, or entire organisms [1,2]. They can be classified into physical and chemical or permanent gels, depending on the nature of the crosslinking points [3]. Molecular entanglements and secondary forces are responsible for the crosslinks in physical gels. This makes them “reversible”, meaning that they can dissolve upon a change of the environmental conditions or just by prolonged exposure to aqueous solutions. Contrariwise, permanent or chemical gels are networks with covalent bonds as crosslinking points.

Hydrogels can be produced either from polymerization and simultaneous crosslinking of hydrophilic monomers in the presence of polyfunctional crosslinking agents, or directly by crosslinking of hydrophilic polymers. Residual amounts of monomers, initiators, catalysts, and their side products can often result in undesirable characteristics, such as color, chemical reactivity, and potential toxicity. Purification is typically performed by extraction into excess water and may take up to several weeks to be completed. Therefore, the demand for facile and safer synthetic approaches makes single component-based processes particularly attractive. 

Irradiation with either gamma rays or accelerated electron beams has been successfully applied to produce hydrogels from water-soluble, biocompatible synthetic polymers, such as poly(acrylic acid) poly(vinyl alcohol), poly(vinylpyrrolidone), poly(ethylene glycol) and polyacrylamide, [4,5] polysaccharides [6,7,8,9], and polyaminoacids [10,11]. One distinct advantage of the recourse to high energy irradiation is that sterilization can also be simultaneously achieved, if suitable doses are imparted. Alternative, reagent-free approaches rely on UV-irradiation of directly photo-crosslinkable polymeric systems, autoclave, or microwave radiation thermal treatments. Direct photo-crosslinking requires the presence of photoactive groups in the polymer, such as coumarin, cinnamic acid, anthracene, or dimethylmaleimide [12,13]. Not many biocompatible water-soluble polymers possess these functional groups. Therefore, it often requires prior ad hoc polymer functionalization. Autoclave or microwave-assisted thermal cross-linking methods are inexpensive and safe, but they do not allow easy control of the locus of reaction, therefore they are not suitable for producing micro-/nanopatterned hydrogel surfaces [14]. 

One further important step towards the obtainment of hydrogels that can accomplish specific roles in biosensing, therapy, or regenerative medicine is the combination of these substrates with biological molecules, such as peptides, antibodies or their portions, oligonucleotides, enzymes, hormones, and several other therapeutic proteins. The incorporated or grafted biological molecules can grant specific functionality [15], while the hydrogel as substrate can provide the biological molecules with an aqueous, three-dimensional microenvironment that can help preserve their structure and functionality [16,17]. 

The possibility of designing and controlling the molecular architecture of hydrogels down to the micro- and nanoscales in the form of patterned surfaces or individual nanoparticles provides further significant advantages over bulk materials towards particular applications in diagnosis and therapy. In this review, Section 2 covers some fundamental aspects of radiation chemistry and physics that are relevant for hydrogel synthesis. Section 3 and Section 4 discuss the strategies and challenges of hydrogel nano-structuring by recourse to highly focused low energy electron beams, to more disperse but highly energetic electron beams, or to γ-photons. Section 5 and Section 6 discuss some applications in the biomedical field.

## 2. Radiation Chemistry and Physics in Hydrogel Technology

Absorption of ionizing radiation such as γ-photons and high energy electrons induces ionization and excitation of matter which subsequently leads to the formation of reactive radicals [18]. The further reactions of the radicals depend on the structure and viscosity of the material and also on the amount of energy deposited in the material. The latter is referred to as the absorbed dose [18]. 

**Figure 1:**
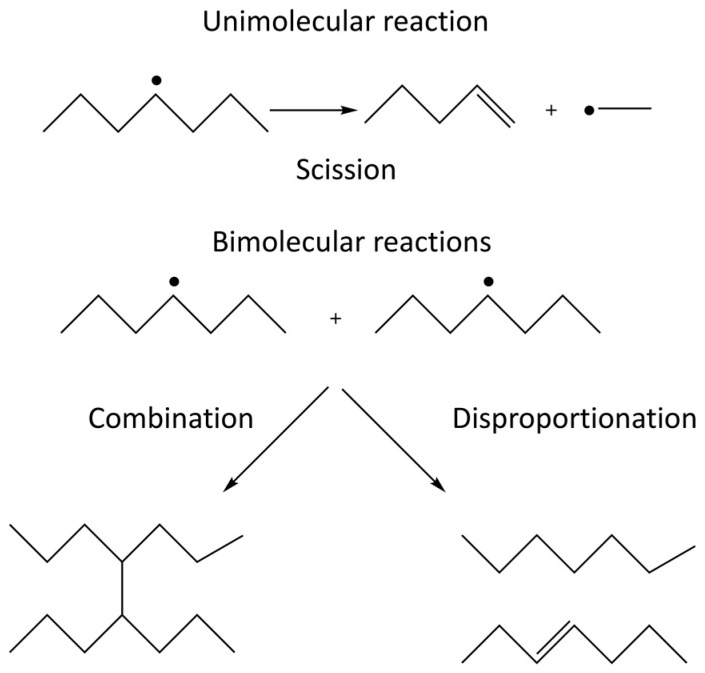
Unimolecular and bimolecular reactions occurring in irradiated polymers. The unimolecular reaction is scission (depolymerization) and the bimolecular reactions are radical-radical combination and disproportionation.

When irradiating a pure polymer, absorption of the radiation energy will lead to the formation of radicals [19]. The radicals formed on the polymer chain can react with radical sites on other polymer chains or on the same polymer chain. These radical-radical reactions can either lead to the formation of covalent bonds, i.e., crosslinking, or to disproportionation where one radical site oxidizes the other radical site. The competition between crosslinking and disproportionation depends on the structure of the radicals involved and cannot be affected by external factors [20]. In addition, the radicals formed can also undergo unimolecular scission (fragmentation) reactions. The competition between the unimolecular scission reaction and the bimolecular radical-radical reaction depends on the structure of the radicals as well as on the concentration of radical sites in the system. The latter is directly influenced by the radiation energy deposited per unit time, also known as the dose rate. In systems exposed to air, the polymer radicals formed may also react with molecular oxygen which will enable additional reactions where chain scission and incorporation of oxidized functional groups are two probable outcomes. The possible reactions in an irradiated polymer system are schematically illustrated in Figure 1 [19,20].

In this paper we primarily focus on crosslinking of hydrophilic polymer chains in order to produce polymer networks that, upon exposure to water, undergo swelling to produce hydrogels. Chain scission and functionalization occur as concurrent reactions.

In systems composed of more than one component, the absorbed radiation energy is distributed between the components according to their relative masses. In aqueous solutions, most of the radiation energy is absorbed by the water. The aqueous radiolysis products are HO^•^, H^•^, e_aq_^−^, H_2_O_2_, H_2_, and H_3_O^+^ [18]. HO^•^ and H^•^ are capable of abstracting H-atoms from most polymers and thereby produce the same types of radicals as the ones that are formed upon irradiation of the pure polymer. However, radicals formed as a consequence of direct scission of the backbone, due to direct absorption of the radiation energy by the polymer, will occur to a much lesser extent in solution. In dilute aqueous polymer solutions, the polymer radical formation can be almost exclusively attributed to the absorption of ionizing radiation by the water and is therefore referred to as an “indirect effect”, while the polymer radical formation in the irradiated pure polymer is referred to as a “direct effect”. 

The polymer radicals formed in aqueous solution can react in the same way as the radicals formed in the pure polymer system. However, the viscosity of the systems as well as the polymer concentration and thereby the polymer radical concentration and spatial distribution differ significantly [18]. This has a major impact on the kinetics of the reactions both in absolute and relative terms. At a sufficiently high polymer concentration, the radical-radical crosslinking reactions will produce a polymer network in the solution, hence a hydrogel [4].

The radiation chemical yields (i.e., the amount of produced or consumed species per absorbed unit of radiation energy) of the aqueous radiolysis products are well known. This enables thorough design of the syntheses in aqueous solution. In general, the radiation chemical yields depend on the type of irradiation and on the radiation energy. However, high energy electrons and γ-photons have the same radiation chemical yields. The radiation chemical yields of the aqueous radiolysis products upon exposure to γ-radiation or high energy electrons are the following (in μmol J^−1^): HO^•^ (0.28), H^•^ (0.062), e_aq_^−^ (0.28), H_2_O_2_ (0.073), H_2_ (0.047), and H_3_O^+^ (0.28) [18]. Interestingly, N_2_O dissolved in water can convert the solvated electron (e_aq_^−^) to a hydroxyl radical and thereby double the radiation chemical yield. For this reason, N_2_O saturation is often employed when performing radiation synthesis of aqueous systems (provided that the hydroxyl radical is the desired reactant).

While γ-photons are in general very penetrating, electrons have a limited penetration depth. The penetration depth of electrons in a given material is strongly dependent on the electron energy where a higher electron energy corresponds to a larger penetration depth [18]. The direct implication of this is that modification of thicker materials requires higher electron energies. For this reason, radiation processing of thin films can be performed with keV electrons, while processing larger volumes of solutions requires MeV electrons. As a consequence, completely different irradiation facilities must be used. 

The transfer of energy from the incident high energy electron to the absorbing material can be quantified in terms of linear energy transfer (LET), which is a measure of absorbed energy per unit path length. The LET value in a given absorber depends on the type of radiation and the energy. In general, the LET value increases with decreasing energy. Therefore, low energy electrons will have a higher LET than high energy electrons. The direct consequence of this is that the distance between ionizations (and thereby radical formation) will be shorter for low energy electrons than for high energy electrons. In other words the radical concentration will be higher and thereby also the probability for radical-radical reactions. 

## 3. Radiation Engineering of Hydrogels at the Micro-/Nanoscale 

### 3.1. Micropatterned Hydrogels by Electron Beam Lithography 

Accelerated electron-beams can be used to create hydrogel patterns at the nano- and micro-length scales on surfaces, with a typical top-down approach. The methodology, known as electron beam lithography (EBL), is a well-established precision tool for nanoscale pattern generation. The polymers are deposited in the form of thin dry films (e-beam resists) on the pre-treated (polished, degreased, etched, and coated with a primer) substrates and subjected to localized irradiation with focused accelerated electron beams of relatively low energy (in the keV range). The pattern is then developed by rinsing the surface with water. This will wash away polymer chains that are not crosslinked or grafted to the substrate. Conversely, crosslinked polymer chains that are also grafted to the substrate will remain on the surface and the network will take up water to yield a hydrogel. EBL has the advantage of being able to generate surface-patterned structures with arbitrary shapes and feature size down to a few tens of nanometers [21,22]. 

The first microscopic hydrogel patterns were created using spatially resolved electron beams from scanning electron microscopes (SEM) and solvent-free polyethylene glycol (PEG or PEO) films as e-beam resist [23,24]. Several further reports describe the fabrication of micro-/nano-gel patterns by EBL using other polymers, such as poly (vinyl methyl ether) [25], poly (vinyl pyrrolidone) (PVP) [26], polyamidoamine [27], oligo(ethylene glycol) methacrylate [28], and star PEGs with functional end groups [29].

It is generally proposed that irradiation of these polymers in vacuum form carbon-centered radicals as a result of the direct effects of irradiation, that can then be involved in several chain-chain and chain-substrate radical reactions. As stated above, chain scission and crosslinking occur simultaneously during electron irradiation, but at different rates depending on the conditions. The overall response of the e-beam resist is thus determined by the dominant of the two competing processes. During the course of irradiation, increased crosslinking will increase the viscosity of the film and thereby reduce the rate constant for bimolecular reactions. Hence, the competition between the processes will change. Conditions to achieve critical crosslinking (i.e., minimum one crosslinking point per chain) and grafting to the underlying surface must be simultaneously fulfilled in order to form stable surface patterned micro-/nanogels. 

**Figure 2:**
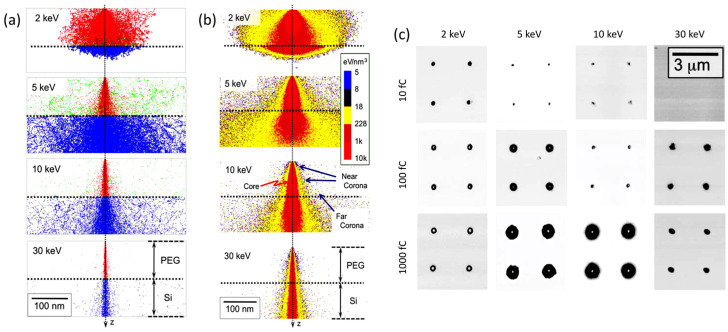
(**a**) Monte Carlo simulation describing the trajectory of 1000 electrons with energies of 2, 5, 10, or 30 keV across a 100 nm thick solvent-free polyethylene glycol (PEG) resist on a Si substrate; primary electrons of the beam are represented in red, scattered electrons in blue, backscattered electrons in green. (**b**) Distribution of deposited energy (**c**). Phase-contrast atomic force microscopy (AFM) images of PEG microgels patterned on silanized Si surfaces with different incident electron energies and different point doses. Based on [30].

When discussing more in detail how it is possible to control the locus of radiation chemical changes of an e-beam resist, it should be recalled that when an accelerated electron impinges on a solid target from vacuum, it undergoes both elastic and inelastic scattering. The nature and extent of these two processes depend on the nature of the absorbent material and initial energy of the incident electron. Electron scattering will have a direct effect on the spatial resolution of EBL. In Figure 2a, the results of a Monte Carlo simulation describing the trajectory of 1000 electrons with energies of 2, 5, 10, or 30 keV across a 100 nm thick PEG resist on a Si substrate are represented [30]. 

Primary electrons (red) that pass through the resist into the underlaying Si layer are scattered to higher angles (blue). Backscattered electrons (green) originate from both media and when generated on Si they can re-emerge into the resist at a large distance from the primary electrons they originated from. This effect, called “proximity effect”, affects the precision of EBL. Indeed, the distribution of deposited energy (i.e., dose distribution) at and around the point of electron-beam incidence can be very non-uniform (see Figure 2b). In high dose regions, called “core” (red, in Figure 2b), crosslinking can be so extensive that the polymer becomes very rigid and not quite gel-like. In the surrounding area, (yellow, in Figure 2b), the absorbed doses are lower and can mainly be attributed to primary electrons scattered from their incident direction (Z) at the top (“near-corona”), and to backscattered electrons at the bottom (“far-corona”). The density of crosslinks of the near-corona is lower and the material upon development can become a soft, hydrated gel. The far-corona has similar gel-like properties to the near-corona but is localized at the interface with the substrate and can extend many tens of nanometers and more away from the core. For low energy electrons, the near-corona can be thick and there can essentially be no far-corona. The reason is that the number of backscattered electrons is low and they have insufficient energy to travel far from the core. In contrast, higher energy electrons can be backscattered from the substrate and can again induce chemical reactions on their second trip through the resist. Yet, the reactions caused by these electrons may not be sufficiently extensive to graft all polymer chains to the network and all network portions to the substrate. Consequently, isolated portions of networks that have formed happen to be removed during development, generating discontinuous layers of gels even at long distance from the core. In some cases, only grafted single chains can be found. In conclusion, the structure and extent of the far-corona is strongly affected by the irradiation conditions. 

Figure 2c shows phase-contrast atomic force microscopy (AFM) images of PEG microgels patterned with different incident electron energies and different exposures. The dark spots correspond to microgels. The white spots in the center of some microgels indicate a difference in mechanical properties of the core with respect to the shell. The different parts of the structure, depending on the degree and density of crosslinking, can have a very different affinity to molecules, biomolecules, and cells. PEG is generally used for its non-adhesive, antifouling properties that minimize non-specific protein adsorption [31], trusting on specific functional groups in PEG chain ends and their reaction with biomolecules for the specific interaction or reaction with the complementary dye, protein, or membrane cell receptor. The hard core might not be hydrated enough to present these “antifouling” properties, but depending on the size of the PEG segments stretching out in the solvent from the loosely crosslinked corona, the core can also be shielded by protein absorption. On the other hand, even a monolayer of PEG is sufficient, for example, to convert a surface from protein-adhesive to protein non-adhesive [30]. Hence, the choice of the electron-exposure conditions for patterning the resist can control the subsequent bio-interactive properties of the micro-/nano-patterned hydrogels over length scales that are relevant to both proteins and cells. 

### 3.2. Radiation Engineering of Hydrogel Nanoparticles

As described above, hydrogels are formed when aqueous polymer solutions are irradiated with ionizing radiation. Under irradiation conditions where the dose rate is high enough to ensure the coexistence of multiple radical sites per polymer chain, the radical-radical reactions can be intramolecular as well as intermolecular. While the intermolecular crosslinking builds a more macroscopic hydrogel, the intramolecular crosslinking can produce hydrogel nanoparticles (nanogels) where the smallest entity is a single polymer chain that has been intramolecularly crosslinked [32]. 

**Figure 3:**
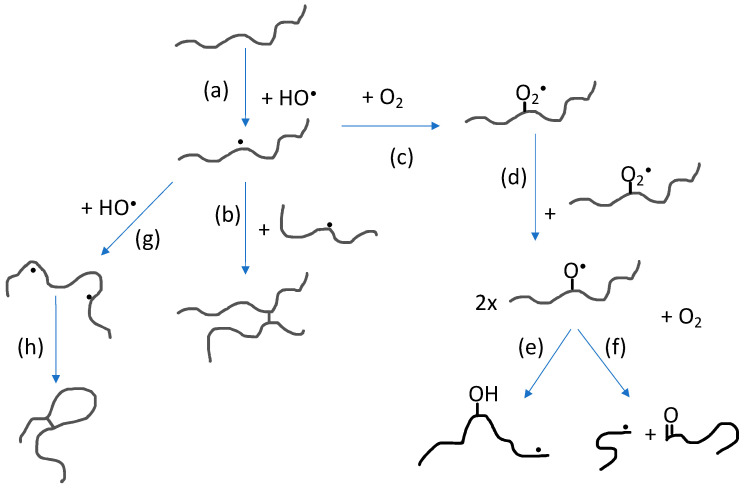
Reactions involving polymer radicals in aqueous solution: (**a**) Hydrogen abstraction by hydroxyl radicals producing polymer radicals; (**b**) intermolecular radical combination (the competing disproportionation is not shown); (**c**) reaction with molecular oxygen; (**d**) reaction between peroxyl radicals; (**e**) intramolecular H-transfer; (**f**) oxyl radical β-scission; (**g**) hydrogen abstraction from polymer radical producing multiple radical sites; and (**h**) intramolecular radical combination (also in this case, disproportionation is not shown).

The impact of polymer concentration and dose rate on nanogel size have been thoroughly investigated [4,5,33,34,35,36]. Intramolecular radical-radical reactions, and thereby also nanogel formation, is favored by low polymer concentration (below the critical chain overlap concentration of the polymer) and high dose rate. High dose rate will promote formation of multiple radical sites per polymer chain while the low polymer concentration will suppress intermolecular interactions in general and thereby also intermolecular radical-radical reactions. When changing the polymer concentration from high to low, the conditions change from favoring formation of macrogels to single chain nanogels. At concentrations below but still relatively close to the critical chain overlap concentration, intermolecular radical-radical crosslinking can initially occur, leading to a decrease in particle concentration after which intramolecular crosslinking becomes dominating. Hence, particle size no longer significantly increases. Extensive intramolecular crosslinking restrains the polymer chain segment mobility and thereby reduces the possibility of radicals to get in close enough proximity to react. This, in turn, progressively reduces the rate of intramolecular radical-radical combination. [33,35] In Figure 3, the reactions involving polymer radicals in aqueous solution are schematically described.

A particular mode of irradiation that has been used in the synthesis of nanogels is the use of pulsed electron beams from electron accelerators. The dose rate during the pulse is usually extremely high but between the pulses there is a relatively long period during which the solution is not irradiated [33,37]. Under these conditions it is more relevant to discuss the dose per pulse rather than the average dose rate. 

As previously mentioned, radical-radical reactions lead to crosslinking as well as disproportionation. The latter reaction does not change the polymer chain length but it introduces alkene functionality on one of the two reacting radical sites according to the general reaction shown in Figure 1. The alkene functionality can be employed in further functionalization of the nanogel.

As mentioned above, by saturating an aqueous polymer solution with N_2_O, the hydroxyl radical yield is doubled. For N_2_O-saturated, dilute polymer solutions, the scavenging of hydroxyl radicals by the polymer is not complete and OH-radical recombination competes with polymer radical formation. This leads to the production of H_2_O_2_ and subsequently also to the formation of molecular oxygen [35,37]. Molecular oxygen reacts rapidly with C-centered radicals, a reaction that will compete with radical-radical reactions and result in oxidation of the polymer. When using PVP as starting material for the nanogel synthesis, it has been shown that primary amino groups as well as carboxyl groups are formed during the irradiation and the number of groups per particle increases with increasing dose [33]. This is a clear advantage when using the nanogels as theranostic nanocarriers in medicine.

## 4. Patterned Hydrogels as Advanced Interfaces with Biological Systems 

Micro-/nanostructured hydrogel patterns have been developed as part of biosensing platforms [38,39] and as advanced substrates for cell cultures [40,41,42] and (bio)chemical syntheses [29]. In the development of protein biochips designed to study protein-protein or protein-cell interactions, patterning properties are important because they allow immobilization of active proteins and/or cells with optimal density and proximity. Furthermore, these soft and hydrated interfaces ensure preservation of protein/cell structure and functionality. By a proper choice of the composition and fabrication conditions, the physico-chemical and mechanical properties of the hydrogel can be optimized and possibly also changed during operation. 

**Figure 4:**
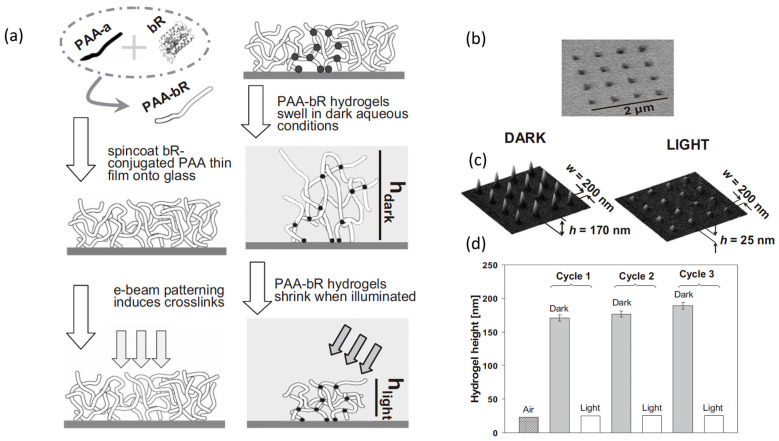
(**a**) Preparation of photo-responsive nanopatterned hydrogels by electron-beam lithography (EBL) using an amine-terminated poly(acrylic acid) conjugated to a protein, bacteriorhodopsin (bR) as resist. bR is a green light-driven proton pump found in the purple membrane of *Halobacterium halobium*. (**b**) SEM image of the hydrogel dot array. (**c**) Contact-mode AFM images of hydrogel arrays after swelling at room temperature in water at pH 9.0. Samples were placed in a fluid cell and imaged before (DARK) and after (LIGHT) illumination by a 514.5 nm light source. (**d**) Average height of the hydrogel dot array in air (patterned bar) and thereafter placed in water at pH 9.0 for three consecutive, cyclic exposures in dark (gray bars) and illuminated conditions (white bars). Adapted from [43].

As shown in Figure 4, light-responsive nanostructured hydrogels have been produced using poly (acrylic acid) (PAA) conjugated to a protein, bacteriorhodopsin (bR), which is a green light-driven proton pump found in the purple membrane of *Halobacterium halobium*. This protein is capable of transporting protons from the cytoplasmic (CP) to the extracellular (EC) side of the cell [43]. The conjugation of the protein to the nanopatterned hydrogel has enabled the obtainment of a light-responsive nanostructured hydrogel that can be used as a smart gate in micro- and nanofluidic devices or as a switchable smart surface for tissue engineering. The protein was first conjugated to PAA, then spin-coated in the form of thin films onto a primed glass substrate and patterned using EBL (see Figure 4a). The focused e-beam of the EBL apparatus induced crosslinking of the irradiated polymer film and network grafting onto the substrate, simultaneously. The non-irradiated parts of the film were dissolved in water and removed leaving 200 nm wide dots, with 500 nm spacing between dots (see Figure 4b). Reversible swelling-deswelling was observed as a result of consecutive exposures to dark and green-light illuminated conditions, which confirms that the incorporated protein was not degraded by irradiation (see Figure 4c,d).

Hydrogel patterning obtained by EBL has been applied to control cell adhesion on surfaces. PEG was cross-linked and grafted on silanized glass substrates to create cell-repulsive barriers in order to confine single bacterial cells in small areas where they can adhere and prevent their proliferation and growth in larger colonies [44].

**Figure 5:**
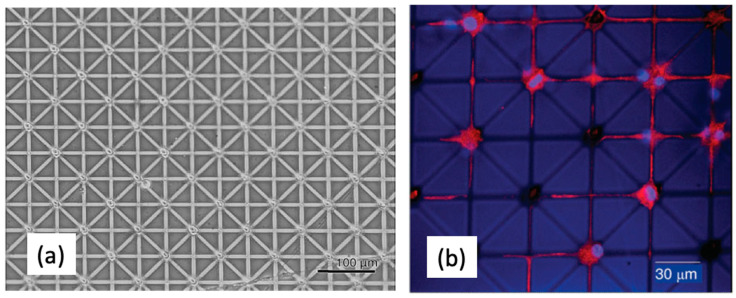
(**a**) Optical transmission microscopy images of a pattern produced with EBL on poly(amidoamine) (PAA) hydrogels. (**b**) Confocal microscopy images of PC12 cells (rat pheochromocytoma) treated with neural growth factor (NGF) (for 48 h), and immunostained with 4′,6-diamidino-2-phenylindole (DAPI, cell nuclei, blue), fluorescein isothiocyanate (FITC) anti-vinculin antibody (focal contacts, red), and tetramethylrhodamine (TRITC) phalloidin (actin filaments, red). Adapted from [27].

With a different approach, amphoteric poly(amidoamine) (PAA) crosslinked films have been first produced and then selectively degraded by exposure to a focused electron beam to generate a pattern [27]. The non-exposed areas were able to be water-swollen to create hydrated regions where proteins and cells did not adhere, whereas the exposed areas could absorb proteins and promote cell adhesion. Depending on the electron energy and deposited dose, the extent and depth of the hydrogel chemical modification—hence density and penetration depth of various proteins—could be controlled. In general, the surface layer was ablated, while deeper zones became richer in amorphous carbon. By changing the hydrogel composition and crosslinking degree, the effect of the e-beam exposure could also be modified. As proof of concept, microwells (10 μm diameter) interconnected by thin channels (1 μm width) were produced to fabricate a structure resembling a neural network, as shown in Figure 5. Figure 5b shows that PC12 cells (rat pheochromocytoma) were able to adhere and grow on the microwells when cultured in the presence of neural growth factor (NGF). The confocal microscopy image evidences that single cell bodies occupy the microcavities, whereas neurites lie in the microchannels. By this approach, the number and location of neurites could be precisely determined by the number and position of microchannels originating by each microwell [27].

**Figure 6:**
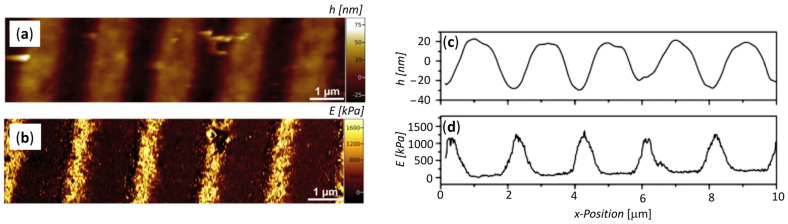
Two-step process to produce a mechanically patterned hydrogel coating, comprising EBL patterning of a dry gelatin film followed by global e-beam irradiation of the subsequently hydrated film. (**a**) AFM topographical image of patterned gelatin layer in the wet state after the two-step process. (**b**) AFM mechanical image showing local E-modulus. (**c**,**d**) Topographical and mechanical profiles of the stripe pattern after global irradiation in the wet state: (**c**) height and (**d**) E-modulus. Adapted from [45].

One major limitation of EBL is the operation under vacuum that requires dry resists and increase the gelation dose. Moreover, it is not applicable to polymer resists that would mainly undergo chain scission under e-beam irradiation. This is the case for gelatin. Therefore, in order to develop substrates with mechanically patterned hydrogels, i.e., substrates with topologically controlled variations of mechanical properties, a two-step process has been proposed which combine “localized” EBL with “global” electron beam irradiation [45]. In other words, the initial stage is based on the direct effect, where the radiation energy is absorbed by the polymer molecules, and the second stage is based on the indirect effect where water absorbs the radiation energy and the aqueous radiolysis products initiate the radical-radical crosslinking. In the first step, the focused electron beam of an ELB system produces a pattern on a dry gelatin film employing lower electron energies (20 keV) and realizing high absorbed dose (approximately 15 MGy). Under these conditions, crosslinking is only expected at the “physical” junction zones of gelatin chains, e.g., around triple helices, whereas fragmentation is expected to dominate otherwise. The rationale for this is that it is only around the triple helices that the polymer chains are sufficiently close to each other for crosslinking to efficiently compete with scission. In the second step, global irradiation with 10 MeV electrons at a dose of 40 kGy was performed in the hydrated state. Under these conditions, the radiolytically produced OH-radicals can abstract hydrogen from the polymer chains leading to the formation of macroradicals, which recombine into the desired crosslinks. As a result of the two step processes, a gelatin layer with precise topographical and mechanical pattern was obtained (see Figure 6).

## 5. Biomedical Applications of Bio-Hybrid Nanogels

As already described in Section 3.2, high energy electrons can be successfully used to produce hydrogel nanoparticles, or nanogels. The success of nanogels is mainly due to their promising applications in the biomedical field. Nanogels, as stand-alone nanoparticles, have been proposed as nanocarriers of chemical and biological entities for therapeutic purposes and/or contrast agents for medical imaging [46,47,48], as active components of biochips or biosensors [49], building blocks of in situ forming scaffolds for tissue engineering, [50,51] cell culture systems [52,53], and antimicrobial coatings [54,55], and as components of wound dressing formulations [56,57]. Other notable applications of nanogels in related fields include iron chelation therapy [58], biochemical separation and contaminant removal [59,60], and bio-catalysis [61]. 

In drug delivery, nanogels can offer some distinct advantages compared to other nano-constructs due to their inherent features, such as high colloidal stability in aqueous media and potentially also in blood, reduced adsorption of plasma proteins and hence prolonged circulatory half-life, flexibility, and possibility to take lateral drift velocity components when moving with the blood stream, favoring extravasation, tunable adhesiveness to epithelial and endothelial cells, sizable drug loading capacity with a variety of loading and controlled release mechanisms, amenability to be combined with lipid cores or shells (nanolipogels) or with inorganic nanoparticles to enable multiple functions, e.g., for chemo-photothermal or chemo-photodynamic cancer therapy, or for image-guided therapies [62].

Nanogel features, such as size, morphology and composition, all impact on nanogels circulation residence time and influence their biodistribution and clearance profiles. While early clearance is not desirable, accumulation at the disease site and degradation or elimination after the delivery function has been accomplished are highly desirable. Several targeting strategies have been proposed and evaluated, that also set requirements on each of the above features of the nanocarrier. The impact of size, surface charge density, and surface chemistry on biodistribution, accumulation at the target site, and clearance have been the topic of several review papers [62,63,64,65,66,67]. One important consideration that emerges is that even subtle changes in the nanogel network can impact the biodistribution and tumor accumulation [68], as well as their cellular internalization mechanism, degree of uptake, and potential for toxicity [69,70].

Although more and more scientific papers describing new nanogel formulations, synthesis methodologies, and potential applications in the biomedical field are appearing, only very few nanogels have already been introduced in clinical trials [71]. Today’s research in nanogel design and development for biomedical applications mainly focuses on how to overcome the restrictions imposed by cost for commercial scale production and on some medical requirements and technological issues for their full exploitation as new platforms in biomedicine.

As already discussed before, the main advantages of using high-energy irradiation to synthesize hydrogel nanoparticles for biomedical applications include minimal recourse to potentially toxic chemicals; simple production schemes; possibility of fine-tuning nanogel size, crosslinking density, and functionality by a proper selection of irradiation conditions, polymer concentration, and composition of the atmosphere (N_2_, N_2_O, air); and the possibility to obtain simultaneous sterilization when the absorbed doses are within the sterilization dose range. One major limitation of nanogel radiation-synthesis is related to the fact that crosslinking cannot be the dominant process for a few classes of potentially interesting hydrophilic polymers, such as polysaccharides and polypeptides, that mainly undergo chain scission under irradiation, but would undergo appropriate biodegradation if used as main components of nanogels. Crosslinking for these polymers may become competitive only upon chemical modification to introduce short alkyl chains and/or by achieving significant local increases of their concentration [72]. In the following sections, some potential biomedical applications of ionizing-radiation engineered nanogels are described.

### 5.1. Nanogels for the Delivery of Therapeutic Molecules to the Brain 

Brain and nervous system disorders represent a large, unmet medical challenge that affects two billion people worldwide; a number that is expected to grow with aging and expanding global population [73,74]. One of the main reasons is the restricted delivery of drugs to the brain due to the presence of formidable obstacles, that are also vital protective barriers for the brain; namely, the blood-brain barrier (BBB) and the blood-cerebrospinal fluid barrier (BCSFB). These barriers prevent the entry of many types of drugs into the central nervous system (CNS). Approaches to deliver drugs to the CNS focus on either locally circumventing the blood-brain barrier (e.g., by intranasal delivery), or to cross it. For the drug to cross the BBB, this needs to be (transiently) opened or, alternatively, passive or active transport mechanisms need to be activated [75,76,77]. The last approach is generally regarded safer than the (transient) opening of the neuroprotective BBB [75].

The work of Rashed et al. 2015 is the first report on the application of radiation-engineered nanogels for drug delivery to the brain. In particular, polyvinylpyrrolidone-poly(acrylic acid) (PVP/PAA) nanogels were developed as carriers of dopamine (DA), a hormone that is used in the medical therapy of Parkinson’s disease (PD) [78]. The nanogels are produced by gamma irradiation (3.7 kGy/h) at dose of 35 kGy from pre-formed PVA/PAA complexes dispersed in water at ambient temperature and air atmosphere. The tasks of the nanogel are to protect DA against in vivo degradation, to improve its pharmacokinetics, and reduce toxicity for peripherical organs. DA was loaded on the nanogels by rehydration in a DA-containing aqueous solution from the freeze-dried powder, with a loading efficiency of ca. 72%. The hydrodynamic mean diameters of free nanogel and DA-loaded nanogel were reported to be about 642 and 572 nm and the surface charge was reported to be positive. Either reserpine or rotenone were administered to rats to generate animal models of PD [79]. In particular, a reserpine-induced model of Parkinsonism was used to evaluate the possible toxicity of the nano-DA after a single or repeated administrations, to determine optimal dose and time of peak efficacy following administration, and to confirm that nano-DA can efficiently deliver DA across the BBB by observing the effect on the Parkinson’s symptoms induced in rats by reserpine injection. The rotenone-induced model Parkinsonism was used instead to conduct behavioral tests (tremor, catalepsy, sensorimotor coordination) and assess eventual neuroprotective effects of the drug. For both types of models, nano-DA was administered by intraperitoneal (i.p.) injection. The behavioral study was complemented by biochemical analysis to measure the striatal DA content and assess eventual mitochondrial dysfunction due to an increase of oxidative stress. The brain concentrations of DA were shown to be remarkably higher in the case of the administration of the drug-loaded nanogels compared to the Parkinsonian rats and at very early time, suggesting that the nano-DA gains access to the brain rapidly upon administration to the animals. The administration of nano-DA induced significant improvements in the catalepsy state of both models of Parkinsonism. The mechanism of transport across the BBB, generically described as “Trojan Horse effect”, is not fully elucidated. Information of biodistribution in the various organs and clearance from the body is also missing.

Another study of ionizing-radiation engineered nanogels for drug delivery to the brain is reported in the two papers that describe the synthesis and characterization of mucoadhesive PVP-g-AA based nanogels designed to deliver biologically active insulin to the brain for the treatment of Alzheimer’s disease by the intranasal route [80,81].

Intranasal delivery can enable drug transport both across the BBB and bypassing the BBB. It can take advantage of the highly permeable vasculature of the nasal mucosa to obtain drug systemic circulation with by-passage of the first-pass metabolism, but it can also access the brain through the olfactory and trigeminal nerves. The axonal transport may involve a slower intraneuronal pathway, which requires internalization in neurons, and a much faster extra-neuronal pathway, where the molecules diffuse through the perineural space surrounding the neurons. Absorption of drugs from the nasal cavity requires though the passage of the drug through the mucus layer and nasal epithelium, that are also challenging biological barriers because of the presence of mucolytic enzymes and tight junctions [75]. Then, a nanogel carrier to deliver insulin to the brain is deemed to perform several tasks; it has to provide mucoadhesive properties, protect insulin from the proteolytic enzymes present in the mucus, reduce anterior leakage, irritation, and damage of the nasal mucosa, and enable insulin interaction with its receptors at the CNS cell membrane, activating the typical biological signals cascade. 

**Figure 7:**
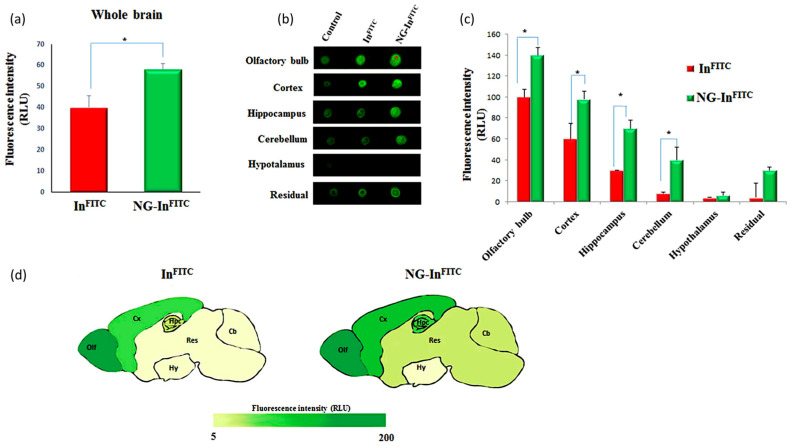
Brain biodistribution of FITC-labelled PVP-g-AA and insulin-conjugated nanogels (NG-In) after 30 min from intranasal (i.n.) administration. (**a**) FITC fluorescence intensity of free insulin (InFITC) (red) and NG-InFITC (green) in the whole brain after the i.n. inoculation of 10 μL per nostril of the two formulations. (**b**) Fluorescence dot spot analysis of different brain areas. (**c**) Distribution of FITC fluorescence signaling levels in the different brain areas of free insulin (red) and NG-In (green). (**d**) Map brain indicating the levels of free insulin (left) and NG-In (right) in the olfactory bulb (Olf), cortex (Cx), hyppocampus (Hpc), hypotalamyus (Hy), cerebellum (Cb) and residual areas (Res). Each data point represents the mean ± SD of n = 5. * *p* < 0.05 vs. indicated groups. From [81].

Nanogel carriers have been produced by pulsed electron-beam irradiation, using a 10 MeV linear accelerator in a typical industrial set up, of semi-dilute PVP solutions with small amounts of acrylic acid (AA) contained in sealed vessels saturated with N_2_O. The integrated dose of ca. 40 kGy was supplied with a single pass [80,82]. The nanogel hydrodynamic diameter is of approx. 50 nm, that is significantly smaller than in [78]. The crosslinked PVP network is functionalized with carboxyl groups derived from grafting acrylic acid but also with primary amino groups derived from radiation-induced chain scission and oxidation reactions, as mentioned before [35]. These functional groups have been used to conjugate insulin (by 1-ethyl-3-(3-dimethylaminopropyl) carbodiimide (EDC) and sulfo N-hydroxysuccinimide (sulfo-NHS) activation of carboxyl groups) and to bind fluorescent probes for both in vitro and in vivo localization studies. The biological evaluation carried out on both naked and insulin-conjugated nanogels (NG-In) has confirmed the absence of toxicity, immunogenicity, and proven excellent hemocompatibility of these nanoparticles [80,81,83]. From in vitro experiments, it seems that insulin in NG-In is protected from protease degradation, is able to activate signaling even if linked to the nanogel with a non-cleavable bond, and is able to exert a neuroprotective effect against the toxicity induced by the amyloid peptide [80]. The whole-body distribution and clearance of the naked nanogel system has been tested on mice after i.p. injection [81]. Nanogels enter the bloodstream (peak after 4 h following administration) and are cleared from the blood in 24 h. Most of the nanogels localize in liver and kidney with a peak after 4 h from administration and are eliminated from all organs in 24 h with a residual 30% in liver and spleen only. Presence of nanogels in the urine has been confirmed. Intranasal (i.n.) administration of naked nanogels has enabled their accumulation in the brain (that was not occurring upon i.p. administration). Interestingly, 30 min after the administration—time that corresponds to the peak concentration for free insulin—NG-In is present in the brain at a higher concentration than free insulin. Nanogels are also present in other organs suggesting that they have entered into systemic circulation. The distribution in the various regions of the brain is shown in Figure 7. The presence of a significant amount of NG-In in the anterior and cerebellar regions of the mouse brain supports a transport mechanism also through the trigeminal and olfactory nerve pathways. The nasal epithelium does not appear damaged. 

### 5.2. Nanogels in Cancer Therapy

The same general experimental approach used to synthetize the PVP-based nanogels for insulin delivery to the brain has been followed by Dispenza and collaborators to generate a family of functionalized PVP nanogels that were conjugated to various targeting ligands and to molecules with potential therapeutic effect in the treatment of solid tumors [83,84,85,86,87,88]. It is well established that solid tumors are characterized by the over-expression of specific antigens or receptors on their cell surface along with newly formed vasculature with a fenestrated endothelium and absent or discontinuous basal membrane [89]. Additionally, fenestrations on tumor vessel endothelium, compared to fenestral diaphragms in other tissues, like pancreas or intestine, have a decreased anionic charge and higher permeability by larger, even negatively charged biomolecules (e.g., albumin) and nanoparticles. Both these features, the overexpression of antigens and/or ligands and the irregular tumor vasculature, can be harnessed to favor the nanocarrier accumulation at the tumor site, penetration into the tumor high pressure interstitial fluid and interaction with the target cells [89]. Alongside the pathophysiological characteristics of the tumor mass, the physicochemical properties and surface chemistry of the drug-loaded nanoparticle control drug targeting. Hence, hydrodynamic diameter and surface charge density are two key parameters [90].

**Figure 8:**
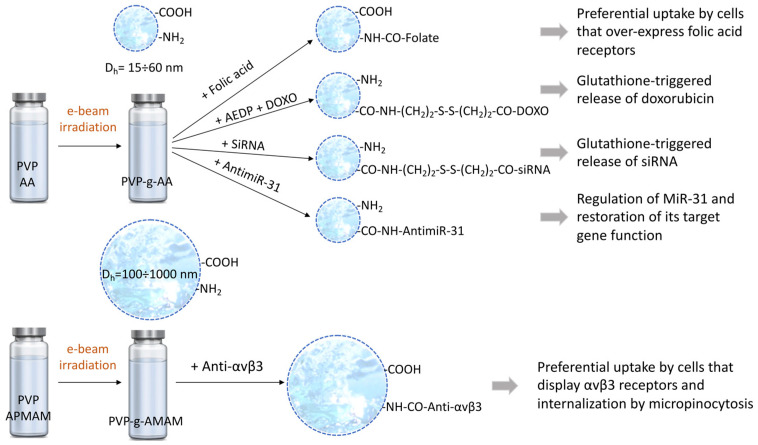
PVP-g-AA and PVP-g-APMAM nanogels produced by e-beam irradiation. The range of average hydrodynamic sizes—obtained by varying polymer concentration, nature of the grafted monomer, and irradiation conditions—are reported. Decoration of the various nanogels with folic acid or Anti-avb3 as targeting ligands, and with potentially therapeutic biomolecules, such as SiRNA, AntimiR-31, or with the cytotoxic doxorubicin were performed. Based on [83,84,85,86,87,88].

Multi-functional nanogels have been synthetized by e-beam irradiation of aqueous PVP solutions in the presence of small variable amounts of either acrylic acid (AA) [82] or 3-N-aminopropyl) methacrylamide hydrochloride (APMAM) [84]. The molar ratio between repeating units in PVP and monomer was varied in the range 50 to 200 and the polymer concentration in the range 0.1%w to 0.5%w. Total doses were generally ca. 40 kGy. Different doses per pulse were applied, yielding different average dose-rates. As expected, nanogel hydrodynamic diameters increased with increasing polymer concentration and decreasing average dose-rate. The addition of AA to PVP produced smaller nanogels (PVP-g-AA), while AMPAM led to bigger nanogels (PVP-g-APMAM) with respect to nanogels obtained from PVP only. These effects have been attributed to the different charges on the two monomers, negative for AA and positive for APMAM, and their impact on PVP macroradical combination. The non-irradiated PVP is slightly anionic due to the keto-enolic tautomerism of pyrrolidone carbonyls. Under irradiation, the anionic character of the forming nanogel increases because of the reactions of macroradicals with forming molecular oxygen and network oxidation. The positively charged APMAM partially shields the negative charges present on the polymer chains favoring radical-radical combination. As a result, for the APMAM containing systems, the nanogel hydrodynamic size steadily increases with dose. This does not happen for AA-containing systems, at least above 20 kGy of dose. All nanogels were characterized by an average negative surface charge density and by the presence of both primary amino groups and carboxyl groups. Either the carboxyl group or the amino group was used to bind bioactive molecules to the nanogel either directly or through a cleavable linker, as sketched in Figure 8. The other functional group has been used to bind a fluorescent label, when required [70].

The folic acid (vitamin B9) decorated variant of a PVP-g-AA, pre-labeled with fluorescein, was incubated in co-cultures of human cervical cancer (Hela) and mouse embryo fibroblast (NIH3T3) cells to demonstrate the preferential uptake from cells that overexpress the folate receptors [85]. The same formulation was used to conjugate either doxorubicin or an amino-terminated siRNA through a heterodifunctional, redox-cleavable linker (namely, 3-(2-aminoethyl) dithiopropionic acid (AEDP)) to demonstrate the ability of the nanogel to release its payload only in cells with increased levels of glutathione, i.e., in response to a stimulus [85,87].

In another study, a PVP-g-AA system was conjugated to a labeled and amino-terminated AntimiR, namely AntimiR-31 [83]. This microRNA inhibitor targets MiR-31, a microRNA overexpressed by primary and metastatic tissue colon cancer cells (CCR) and target of the E2F2 gene. This gene plays a crucial role in the control of CCR progression and in the action of tumor-suppressing proteins. Administration of AntimiR-31 aims to restore the E2F2 gene function. Direct administration of AntimiRs is not a viable strategy because of their enzymatic instability and unfavored internalization. In the proposed approach of ref [83], similarly to the insulin-nanogel conjugates, the oligonucleotide was bound directly to the nanogel through an amidic bond. The NG-AntimiR-31 (Dh ~ 50 nm and ζ-pot ~ −40 mV) was incubated with a colon cancer cell line from lymph node metastasis. The ability to be internalized, to down-regulate the expression of MiR31, and to restore the E2F2 gene function were demonstrated. Finally, a PVP-g-APMAM nanogel variant was conjugated with a monoclonal antibody which recognizes the αvβ3 integrin on activated endothelial cells with the aim to elucidate the specific internalization pathway of these immuno-nanogels [88]. Micropinocytosis was demonstrated to be the main entry mechanism.

### 5.3. Nanogels for Ophthalmic Applications

Nanogels based on PAA/PVP complexes were synthesized by gamma irradiation and evaluated in ophthalmic applications by El-Rehim and collaborators [25,91]. One study concerns the use of the nanogels as carriers of pilocarpine, a molecule that is used for the topical treatment of open-angle and other chronic glaucoma [25]. The major problem of administering pilocarpine is that the molecule is subjected to hydrolytic degradation at neutral pH, whereas it has better chemical stability but low ocular absorption at pH between 4 and 5. This results in low ocular bioavailability and the need for repeated administrations, with associated risks of low patient compliance and side effects. Poly(acrylic acid) is a mucoadhesive polymer that could potentially enhance both the chemical stability of pilocarpine and its adsorption, but it is an irritant for the eye. Moreover, the high viscosities of the PAA solutions cause blurred vision and crystal formation on lids and lashes. The inclusion of PAA as a component of chemically crosslinked PVP/PAA nanogels incorporating the cationic pilocarpine drug was demonstrated to be a viable strategy to develop a low viscosity simulated tear fluid with a therapeutic potential for glaucoma. PAA was synthetized from AA by gamma irradiation (dose rate of 3.7 kGy/h, doses of 20 kGy and 40 kGy) in the presence of PVP acting as templating polymer, varying the molar ratio between PVP’s repeat units and AA, and the total concentration of PVP and AA in water. Polymerization of AA and simultaneous complexation between the formed PAA and PVP, provided that a critical chain length for PAA was reached, led to stable aggregates and mutual crosslinking. The nanogel particles produced were characterized by hydrodynamic diameters varying between approximately 80 nm and 300 nm at pH 4 (when they are in the shrunk state) with up to four-times size increase at pH 7 (when they are in the swollen state, due to the shift of the acid-base equilibrium of the carboxyl groups towards the anionic form that destabilizes interpolymer complexation and causes chain-chain repulsion). As expected, the nanogels were characterized by negative surface charge densities (ζ-potential in the range −20–−30 mV). Pilocarpine loading was carried out by mixing the drug with the aqueous dispersion of the nanogels, relying on electrostatic binding interactions and with a loading efficiency that reached a maximum of ca. 50%, for the nanogels obtained from solutions with the highest AA content irradiated at the highest doses. The incorporated pilocarpine was slowly released at pH 6 and body temperature. Nanogel dispersions resulted transparent and low viscosity enough to be qualified as artificial tears, non-irritating, and mucoadhesive. The same nanogels were tested as low-viscosity artificial tears for the dry eye syndrome treatment on an animal model of the syndrome and their performance compared with an established commercial product. A faster curing of the dry eye signs was observed and interpreted as the result of improved adsorption and retention of the nanogels at the cornea-tear interface [91].

## 6. Conclusions

Hydrogels are increasingly considered key components in the development of biomedical micro-/nanodevices for sensing, drug delivery, and tissue engineering. Their tunable architecture and therefore adjustable hydrophilicity/amphiphilicity, softness, and degradability, together with their high loading capacity for guest biomolecules, make them a highly convenient material platform, while mitigating the risks of cytotoxicity, immuno-response and interference with the biological function. Micro-/nanostructured hydrogels will surely have a role in the development of implantable, flexible sensing, and drug dispensing microdevices, microelectrodes, or neural probes. Nanogels are already involved in pre-clinical studies as therapeutic drug carriers and multimodal imaging agents. Today’s research focuses also on how to overcome the restrictions imposed by cost for commercial scale production. In this respect, electron irradiation can give an important contribution. The possibility of fabricating hydrogels with no recourse to potentially toxic chemicals is reflected in simple production schemes and better product quality. The possibility of fine-tuning the hydrogel structure, shape, and properties only by a proper selection of irradiation conditions (electron energy, dose-rate, dose, vacuum or gaseous atmosphere) is a very attractive prospective. Another potential advantage is the attainment of simultaneous sterilization, when the absorbed doses are within the sterilization dose range. However, there are still some limitations to overcome. One is related to the fact that crosslinking cannot be the dominant process for classes of potentially interesting hydrophilic polymers, like many polysaccharides and proteins that mainly undergo chain scission under irradiation. These macromolecules would be interesting substrates because they can possibly undergo appropriate biodegradation if used as main components of the hydrogels and show inherent bioactivity. Crosslinking of these biomacromolecules becomes competitive only upon chemical modification by introducing radiation-crosslinkable short alkyl chains or by achieving significant local increases of their concentration, like when densely aggregated domains form. To achieve this, the synergistic combination of two types of structures, such as those that derive from self-assembly of biomacromolecules, and those that are generated by radiation-induced crosslinking or scission, has the potential to produce new materials that possess high levels of structural organization together with controlled morphology at the nanoscale and novel tailor-made properties.

## Data Availability

Data sharing not applicable.
